# Herceptin® (trastuzumab) in HER2-positive early breast cancer: protocol for a systematic review and cumulative network meta-analysis

**DOI:** 10.1186/s13643-017-0588-2

**Published:** 2017-10-10

**Authors:** Florence R. Wilson, Megan E. Coombes, Quinlan Wylie, Mariya Yurchenko, Christine Brezden-Masley, Brian Hutton, Becky Skidmore, Chris Cameron

**Affiliations:** 1 Cornerstone Research Group, Inc., Suite 204, 3228 South Service Road, Burlington, Ontario L7N 3H8 Canada; 2 F. Hoffmann-La Roche Ltd, Mississauga, Ontario Canada; 3 St. Michael’s Hospital, Toronto, Ontario Canada; 4 Ottawa Hospital Research Institute, Ottawa, Ontario Canada; 5 Public Health and Preventative Medicine, University of Ottawa School of Epidemiology, Ottawa, Ontario Canada; 6 Independent information specialist, Ottawa, Ontario Canada

**Keywords:** Early breast cancer, HER2-positive breast cancer, Herceptin®, Targeted therapy, Trastuzumab, Survival, Systematic review, Network meta-analysis

## Abstract

**Background:**

Human epidermal growth factor receptor 2-positive (HER2+) breast cancer is an aggressive disease that makes up about 20% of all invasive breast cancers. HER2+ breast cancer is associated with poor prognosis and high mortality rates, but the development of HER2-targeted therapies, such as originator trastuzumab (Herceptin®), has substantially improved patient survival. Numerous clinical trials and reviews have investigated the efficacy of HER2-targeted therapies over the past few decades; however, no study has specifically investigated the vast body of evidence on trastuzumab in comparison to chemotherapy regimens, endocrine therapies, and other targeted therapies. This systematic review and cumulative network meta-analysis (NMA) will synthesize available evidence to evaluate the survival benefit conferred by the addition of originator trastuzumab to standard chemotherapy and to compare the most widely used trastuzumab regimens in patients with HER2+ early breast cancer, based on results from randomized controlled trials (RCTs) and comparative observational studies.

**Methods/design:**

A systematic search of Embase, MEDLINE®, and the Cochrane Library has been designed by an experienced medical information specialist and peer reviewed by another senior information specialist. RCTs and comparative observational studies of patients with HER2+ early breast cancer indexed from 1990 onwards will be eligible for inclusion. Two investigators will independently assess studies for inclusion and use standardized data extraction templates to collect data on study and patient characteristics. The primary outcome of interest is overall survival. Bayesian cumulative NMA methods will be used to quantify the evolution of publicly available evidence using both fixed and random effects models.

**Discussion:**

This study will evaluate survival trends associated with originator trastuzumab in patients with HER2+ early breast cancer. As originator trastuzumab has been researched in both clinical and real-world settings for close to 20 years, a cumulative NMA is likely to show improved precision around the parameter estimates for trastuzumab now compared with when the drug was initially launched in the USA in 1998. A better understanding of the evolution of publicly available comparative evidence for originator trastuzumab will further inform treatment for patients with HER2+ early breast cancer, providing benefit to patients, health professionals, and researchers.

**Systematic review registration:**

PROSPERO CRD42017055763 https://www.crd.york.ac.uk/PROSPERO

**Electronic supplementary material:**

The online version of this article (10.1186/s13643-017-0588-2) contains supplementary material, which is available to authorized users.

## Background

Breast cancer is one of the most common types of cancer, making up about 25% of all cancer diagnoses in 2012 [1]. Advances in treatment and diagnostic techniques have drastically lowered mortality rates; however, breast cancer remains the second leading cause of death for women [2]. Human epidermal growth factor receptor 2 (HER2) is a transmembrane tyrosine kinase that controls cellular division and repair in breast cells. The overexpression of HER2, termed HER2-positive (HER2+), can result in uncontrolled growth and division of breast cells [3]. Approximately 20% of all invasive breast cancers are HER2+, which is a particularly aggressive form of the disease [4, 5]. Before current treatment options became available, only 2–5% of HER2+ breast cancer patients were classified as “long-term survivors” [4]. Fortunately, HER2-targeted therapies have been developed beginning with originator trastuzumab (Herceptin®; F. Hoffmann-La Roche Ltd.) in the 1990s to help combat this aggressive cancer.

In 1998, the US Food and Drug Administration (FDA) approved originator trastuzumab (Herceptin®) as the first antibody-targeted therapy for breast cancer [6]. Health Canada followed suit in 1999 and approved originator trastuzumab (Herceptin®) for the treatment of metastatic breast cancer [7]. Soon after, the FDA and Health Canada expanded the approved use of originator trastuzumab for the treatment of early stage HER2+ breast cancer following the promising results of adjuvant breast cancer clinical trials [8, 9]. Trastuzumab binds to the extracellular domain IV of HER2, thereby inhibiting downstream cell signaling that is implicated in cell proliferation, survival, motility, and adhesion [10]. Clinical trials in HER2+ breast cancer have established that treatment with originator trastuzumab in combination with chemotherapy, compared with chemotherapy alone, increases the time to disease progression and overall survival (OS) in both the metastatic and adjuvant settings [11–15].

Many clinical trials have investigated the efficacy and safety of originator trastuzumab over the past few decades, including NSABP B-31, NCCTG N9831, BCIRG-006, and the Herceptin® Adjuvant (HERA) trials. The NSABP B-31/NCCTG N9831 joint analysis and the BCIRG-006 clinical trial investigated the use of originator trastuzumab in combination with standard adjuvant chemotherapies, such as doxorubicin, cyclophosphamide, and paclitaxel, compared with standard chemotherapy alone [16, 17]. In both cases, treatment with originator trastuzumab significantly improved disease-free survival (DFS) and OS in HER2+ early breast cancer patients [16, 17]. The HERA trial was a phase III randomized controlled trial (RCT) that investigated the efficacy of originator trastuzumab administered for 1 or 2 years in combination with standard chemotherapy, compared with standard chemotherapy alone [18]. The initial trial results were overwhelmingly positive, and patients assigned to chemotherapy alone were allowed to receive originator trastuzumab [18]. At a median follow-up of 8 years, DFS and OS were significantly improved for patients who received 1 year of trastuzumab compared with chemotherapy alone, although neither DFS nor OS differed between the trastuzumab groups [18]. These key trials suggest that originator trastuzumab is a highly efficacious treatment for patients with HER2+ early breast cancer.

While originator trastuzumab is used worldwide, there are a number of other HER2-targeted therapies that have since been developed and used successfully, including lapatinib, pertuzumab, and trastuzumab emtansine. Several systematic literature reviews (SLRs) and meta-analyses (MAs) have been conducted to evaluate the safety and efficacy of HER2-targeted therapies. A recent systematic review by Nagayama et al. [19] searched MEDLINE® and the Cochrane Central Register of Controlled Trials for RCTs published up to August 2012. Eligible studies contained at least two treatment arms, including chemotherapy and/or an anti-HER2 agent in patients with pre-operative HER2+ breast cancer. Outcomes were analyzed from 10 RCTs, and a Bayesian network meta-analysis (NMA) was conducted to investigate the effect of different neoadjuvant therapies on pathologic complete response (pCR). Findings from this NMA suggest that the combination of originator trastuzumab and pertuzumab with neoadjuvant chemotherapy is more effective than chemotherapy and originator trastuzumab alone [19]. Outcomes were significantly worse for chemotherapy and lapatinib than for chemotherapy and originator trastuzumab [19]. An NMA consists of a network of multiple comparators (i.e., interventions to treat the same disease) to assess how they compare in achieving a certain outcome (e.g., patient survival). The advantage of using an NMA instead of a more conventional MA is that the network allows indirect comparisons to be made between interventions which did not exist in the primary research.

While studies such as that by Nagayama et al. [19] are invaluable to our understanding of anti-HER2 therapies in treating breast cancer, no study has yet been carried out to specifically investigate the vast body of publicy available evidence on originator trastuzumab and how this evidence has changed over time. We will perform an SLR and cumulative NMA to investigate the survival advantage conferred by the addition of originator trastuzumab to standard chemotherapy and also to compare the most widely used trastuzumab regimens in HER2+ early breast cancer. This will serve to quantify the value of decades of research on originator trastuzumab and to further define its benefit to patient survival.

## Methods

### Study registration

This protocol is registered on PROSPERO (CRD42017055763), https://www.crd.york.ac.uk/PROSPERO, and is designed to identify and summarize the published comparative data on originator trastuzumab relative to existing treatments on survival outcomes in HER2+ early breast cancer. This protocol has been designed and reported according to the Preferred Reporting Items for Systematic Review and Meta-Analysis Protocols (PRISMA-P) guidelines [20] (Additional file 1).

### Search strategy

An experienced medical information specialist developed and tested the search strategy using an iterative process in consultation with the review team. Another senior information specialist peer-reviewed the strategy prior to its execution using the PRESS checklist [21] (Additional file 2). Using the OVID platform, we will search Embase and Ovid MEDLINE®, including Epub Ahead of Print and In-Process & Other Non-Indexed Citations. We will also search the Cochrane Library on Wiley. Monthly alerts will be established. Strategies will use a combination of controlled vocabulary (e.g., “Breast Neoplasms,” “Chemotherapy, Adjuvant,” “Trastuzumab”) and keywords (e.g., “HER 2,” “adjuvant chemotherapy,” “Herceptin”). Vocabulary and syntax will be adjusted across databases. Standardized filters will be applied for study designs, including the Cochrane highly sensitive search strategy for RCTs. Results will be limited to the English language and publication dates January 1, 1990, to present. Although the first originator trastuzumab trial was initiated in 1992 in metastatic breast cancer, the first adjuvant originator trastuzumab trial was not published until 2005 [12, 22]. Therefore, we chose 1990 as our starting period to ensure the capture of all relevant studies.

In addition, we will perform a targeted gray literature search of trial registries using ClinicalTrials.gov, and we will review bibliographies of relevant SLRs and MAs identified via the database searches. These targeted searches will allow us to cross-reference our study list with registered clinical trials, and existing reviews to ensure that no studies are missed. Specific details regarding the proposed search strategies appear in Additional file 3.

### Eligibility criteria

Studies that meet the following PICOS (Population-Intervention-Comparators-Outcomes-Study design) criteria will be included in this review:

#### Population

Studies involving adult patients (≥ 18 years) with HER2+ early breast cancer (stages 0 to IIIC) will be included. Patients with locally advanced or inflammatory breast cancer and patients receiving neoadjuvant and adjuvant therapies will be included.

#### Intervention

The intervention of interest in the NMA will be originator trastuzumab administered intravenously (IV) in the early breast cancer setting. All trastuzumab doses, treatment schedules, and durations will be eligible. Trastuzumab may be combined with any other drug regimen.

#### Comparators

Various drug regimens for HER2+ early breast cancer that do not include trastuzumab IV will be included as comparators, including chemotherapy agents (e.g., carboplatin, docetaxel, epirubicin), hormonal therapies (e.g., anastrozole, fulvestrant, tamoxifen), and targeted therapies (e.g., bevacizumab, lapatinib, trastuzumab emtansine). All doses, formulations, and treatment durations will be eligible. All included comparators are provided in Additional file 3.

#### Outcomes

Overall survival is the primary outcome of interest. If possible, we may also evaluate at least one measure of x-free survival (xFS), where x stands for measures such as disease (DFS), invasive disease (iDFS), event (EFS), and recurrence (RFS). The choice of xFS outcome will be based on data availability and homogeneity of outcome definitions across studies. If possible, pCR will also be evaluated. Median endpoints and hazard ratios (HR) will be extracted for each outcome, when available.

#### Study design

Randomized controlled trials and comparative observational studies (e.g., case-control, cross-sectional, longitudinal, and cohort studies) will be included. Any comparative observational studies that meet our inclusion criteria will be potentially eligible for inclusion, including but not limited to studies using propensity score methods or multivariable regression, provided that hazard ratios that appropriately adjust for covariates are available.

### Study screening

Study screening will be conducted by two reviewers who will independently review the study records, citation titles, and abstracts identified in the literature search to assess study eligibility based on the PICOS criteria. Reviewers will document reasons for exclusion and present the results in the form of a PRISMA flow diagram [23]. Citations considered to describe potentially eligible articles will be independently reviewed in full-text form for formal inclusion in the final review. Disagreements will be resolved by discussion or by an independent third reviewer not involved in the data collection process.

### Data extraction

Details for selected articles will be collected using standardized data extraction templates, including general study information (trial name, author, publication date, NCT number), study characteristics (study design, blinding, setting, interventions, dosing regimens, treatment duration, length of follow-up), baseline population characteristics (sample size, age, gender, Eastern Cooperative Oncology Group (ECOG) performance status, disease status [stage, hormone receptor status, HER2 status], previous treatments/surgeries, presence of risk factors), definition of survival measures, results, assessment of risk of bias by outcome, and study limitations (compliance, cross-over). For non-randomized studies, additional details outlined in STROBE Guidelines will be captured [24]. For example, we will capture information related to study design, statistical methods and analysis (including those used to control for confounding), covariates considered, data sources, methods used to examine subgroups and interactions, how missing data was addressed, confounder-adjusted estimates, unadjusted estimates and their precision, and numbers of individuals at each stage of the study (numbers potentially eligible, examined for eligibility, confirmed eligible, included in the study, completing follow-up, and analyzed). An independent reviewer will review the data extraction document to check data accuracy and will document quality review throughout. No adverse events/safety information will be extracted as per protocol; therefore, the study report and final publication will not include a summary of adverse events.

### Risk of bias assessment

The risk of bias assessment of eligible studies will be performed in duplicate using the Cochrane Risk of Bias Assessment Tool for Randomized Controlled Trials [25] and the Cochrane Risk Of Bias in Non-randomized Studies - of Interventions tool (ROBINS-I) [26]. The results will be reported as summary tables in the final analysis to highlight any weaknesses in the studies and to help us address any discrepancies in results.

### Approach to evidence synthesis

Based on the findings of the SLR, Bayesian NMAs will be conducted to calculate the effect of Herceptin® on the survival outcomes of interest, based on well-established methods by the National Institute for Health and Care Excellence (NICE) [27, 28]. We will perform a cumulative NMA to illustrate the survival advantage conferred by the addition of originator trastuzumab to standard chemotherapy and also to compare the most widely used trastuzumab regimens. Studies selected for inclusion will be reviewed to assess the distribution of treatment effect modifiers across studies and to assess the validity of the assumptions of homogeneity, similarity, and consistency [29].

#### Cumulative network meta-analysis methods

A cumulative meta-analysis is a series of meta-analyses sequenced according to the chronology of the publication date of included trials, wherein each meta-analysis in the series incorporates additional studies over time; however, most cumulative meta-analyses to date have focused on only comparing two treatments (i.e., traditional meta-analysis). A cumulative NMA will be used to evaluate networks of originator trastuzumab over time. This method will be particularly beneficial to quantify the value associated with the years of clinical experience and publicly available information about the survival benefit conferred by originator trastuzumab in patients with HER2+ early breast cancer. As originator trastuzumab has been researched in both clinical and real-world settings for close to 20 years, it is likely to have greater precision around its parameter estimates now compared with when the drug was initially launched in the USA in 1998, and this can be clearly reflected using a cumulative NMA rather than a standard NMA.

#### Evidence network geometry

Evidence networks will be constructed to best reflect the interventions of interest, based on advice from clinical experts. To maximize clinical relevance, therapy doses that are importantly different will be separated into distinct nodes, while other similar treatments will be pooled together. Separate evidence networks will be generated over time and separate NMAs will be conducted based on survival outcomes of interest, publication date, and study design.

#### Planned methods of analysis and summary measures of treatment effect

Both fixed effects and random effects NMA models will be conducted. A cumulative NMA using the random effects model will be used as the reference case. Vague or flat priors, such as N(0, 100^2^), will be assigned for basic parameters throughout, although informative priors will also be considered. A normal likelihood model which accounts for use of multi-arm trials will be used for analyses. In accordance with NICE Technical Support Document (TSD) methods [27, 28], the log HR will be treated as a continuous outcome and the final results will be subsequently exponentiated. As a measure of the association between each treatment and its efficacy, Markov Chain Monte Carlo methods will be used to model HR point estimates and 95% credible intervals (CrIs) for each pairwise comparison for survival outcomes of interest. Time permitting, we will also run NMAs analyzing whole survival curves as a sensitivity analysis, whereby a multi-dimensional treatment effect approach will be used to model the hazard over time with fractional polynomials [30–33]. The cumulative NMA will focus on pairwise comparisons between the two most widely used trastuzumab regimens and a reference treatment. We will generate “probability better” values as a measure of effect to show the probability of one treatment regimen being better than another within each pairwise comparison of interest.

All analyses will be conducted using R (R Core Team, Vienna, Austria) and WinBUGS software (MRC Biostatistics Unit, Cambridge, UK) based on the WinBUGS code outlined in the NICE Evidence Synthesis TSD Series [27, 28]. Model convergence will be assessed using trace plots, the Brooks-Gelman-Rubin statistic, and inspection of Monte Carlo errors [27]. Three chains will be fitted in WinBUGS for each analysis, with at least 40,000 iterations, and a burn-in of at least 40,000 iterations.

#### Assessment of heterogeneity and inconsistency

A key assumption behind NMA is that the analyzed network is consistent; that is, there is no conflict between direct and indirect evidence. We will assess inconsistency using methods outlined in NICE Evidence Synthesis Technical Support Series [34]. Specifically, assessment of consistency will be based on assessing model fit using the deviance information criterion (DIC) and comparison of the posterior residual deviance from each NMA to the corresponding number of unconstrained data points (approximately equal if fit is adequate) [34]. Scatterplots of deviance residuals and consistency versus inconsistency estimates for each outcome will be inspected to identify potential studies contributing to inconsistency. Additionally, NMA results will be qualitatively compared with direct frequentist pairwise estimates.

Network meta-analysis requires that studies are sufficiently similar in order to pool the results. Exchangeability is a key assumption underlying NMA, and additional concerns arise when RCTs and non-randomized studies are both included [35]. Including high-quality non-randomized studies can allow larger, diverse populations, and additional treatments to be included; however, including low-quality non-randomized studies can introduce confounding bias if the baseline characteristics and risk factors are substantially different between treatment groups [35–37]. Based on these factors, available study and patient characteristics will be thoroughly assessed to ensure similarity and to investigate the potential impact of heterogeneity on effect estimates. Depending on data availability, clinical and methodological heterogeneity will be assessed, and sensitivity, subgroup, and meta-regression analyses will be conducted where possible. Group-level factors will also be considered, such as neoadjuvant investigational therapy, adjuvant investigational therapy, treatment with anthracycline-based chemotherapy, treatment with non-anthracycline-based chemotherapy, node positive breast cancer, node negative breast cancer, hormone receptor-positive (HR+) breast cancer, hormone receptor-negative (HR-) breast cancer, small (< 2 cm) tumor size, and large (≥ 2 cm) tumor size.

#### Network meta-analysis methods for incorporating non-randomized studies

There are various approaches for combining RCTs and non-randomized studies in NMAs, and the validity of the studies must be carefully evaluated [35, 38–41]. For analyses or sensitivity analyses incorporating non-randomized studies, we will use a Bayesian hierarchical model, which is generally considered the most flexible [38–41]. A Bayesian hierarchical model is a statistical model that estimates the parameters of the posterior distribution using the Bayesian method [38–41]. In the model, a study design level (e.g., RCT, non-randomized study) is introduced [38–41]. This approach allows for bias adjustments, as well as a direct comparison of study design-specific estimates to overall estimates. For example, evidence from individual studies of the same design can first be combined to produce study design level estimates; the study design level estimates can then be combined to obtain overall estimates [38–41]. It also gives an estimate of consistency between study designs. NMA results from the Bayesian hierarchical model will be stratified by RCTs alone, by non-randomized studies alone (if possible), and by combining RCTs and non-randomized studies. Stratification on various non-randomized study designs (e.g., case-control, cross-sectional, longitudinal, cohort studies) and statistical analyses for certain study designs (e.g., propensity score matching, disease risk scores, multivariable regression) will also be considered.

## Discussion

The development of HER2-targeted therapies, such as trastuzumab, has been the key for treating HER2 overexpressing cancers which were previously associated with high relapse and mortality rates. Since the FDA approval of originator trastuzumab (Herceptin®) in 1998, other drugs have been approved which similarly act on the HER2 tyrosine kinase. After decades of research on originator trastuzumab and now that there are various therapy options available for HER2+ breast cancer, the oncology field would benefit from a large-scale appraisal of the body of evidence. To our knowledge, we are the first to carry out such a large-scale evaluation of the survival advantage of originator trastuzumab in comparison to chemotherapeutic regimens, endocrine therapies, and other HER2-targeted therapies in the curative setting. Our decision to include both RCTs and non-randomized studies makes this NMA unique as most NMAs do not include comparative observational studies. While this complicates the Bayesian analysis, including non-randomized studies will provide more data on competing therapies and will expand the evidence network to provide more evidence to strengthen the comparisons. Additionally, observational studies may more accurately reflect the realities of the patient experience as they navigate the medical field.

The primary outcome of interest will be overall survival, typically measured as the percentage of patients in a given treatment group who survive to the end of the study or measured from the time of randomization to the time of death from any cause [42]. The selection of OS as the primary endpoint was a strategic choice based on several factors. Importantly, OS is a universal measurement that directly evaluates the benefit of a given treatment and thus is traditionally considered to be the most clinically relevant endpoint. It is an objective endpoint that is easily measured and consistently defined across studies, and therefore rarely subject to error [42].

If possible, we may also evaluate at least one measure of pCR or xFS, including DFS/iDFS, EFS, or RFS. These endpoints provide important measurements for clinical outcome in patients. The use of xFS in analyses relies on data availability and homogeneity of outcome definitions across studies; however, there is variability in the definitions of each of these outcomes. For example, DFS can be reported as time passed until symptoms of cancer arise, as the FDA and National Cancer Institute both define it [43], whereas other institutions such as Cancer Research UK report DFS as the proportion of patients who are alive and cancer-free after a specified amount of time, usually a year [44]. Additionally, there are inconsistencies in what defines a breast cancer event, such as instances where in situ carcinomas could include both lobular and ductal cases or ductal cases alone [45]. There are also inconsistencies in what defines a secondary cancer (i.e., if it includes contralateral breast cancer, excludes non-breast cancers or unknown cancer at non-breast sites) [45]. There is also a greater chance of missing or incomplete data for xFS endpoints, which may lead to biased results due to censoring [46, 47]. Based on these reasons, our analyses will focus primarily on OS.

The current study will be the first systematic review and cumulative NMA that specifically evaluates the totality of the publicly available evidence on originator trastuzumab as a treatment for HER2+ early breast cancer, and the first NMA that combines RCTs and non-randomized studies of originator trastuzumab compared with alternative treatments. These analyses will be an important contribution to the field, which needs a comprehensive summary of the evolution of publicly available comparative evidence for the survival benefit conferred by originator trastuzumab. By investigating the survival advantage conferred by the addition of originator trastuzumab to standard chemotherapy regimens and by comparing the most widely used trastuzumab regimens, we will further inform the treatment of patients with HER2+ early breast cancer.

## Additional files

Additional file 1: PRISMA-P Checklist. Contains the completed PRISMA-P checklist. (DOCX 31 kb)

Additional file 2: PRESS Checklist. Contains the completed PRESS checklist. (DOCX 80 kb)

Additional file 3: Search Strategy. Contains the search strategy to be used for the planned systematic literature review. (DOCX 37 kb)

## Abbreviations

CrI: Credible interval
DFS: Disease-free survival
ECOG: Eastern Cooperative Oncology Group
EFS: Event-free survival
HER2: Human epidermal growth factor receptor 2
HR: Hazard ratio
HR−: Hormone receptor-negative
HR+: Hormone receptor-positive
IDFS: Invasive disease-free survival
IV: Intravenous
MA: Meta-analysis
NMA: Network meta-analysis
OS: Overall survival
pCR: Pathologic complete response
PRISMA: Preferred Reporting Items for Systematic Reviews and Meta-Analysis
RCT: Randomized controlled trial
RFS: Recurrence-free survival
ROBINS-I: Risk Of Bias in Non-randomized Studies - of Interventions
SLR: Systematic literature review
xFS: x-free survival
