# Supplementary material for: Herceptin® (trastuzumab) in HER2-positive early breast cancer: protocol for a systematic review and cumulative network meta-analysis
Source: Syst Rev. 2017 Oct 10;6:196. doi: 10.1186/s13643-017-0588-2 (PMC5634826; doi:10.1186/s13643-017-0588-2)
Supplement: Supplementary file 2 — PRESS Checklist. Contains the completed PRESS checklist. (DOCX 80 kb) [file 13643_2017_588_MOESM2_ESM.docx]

**Additional file 2: PRESS Checklist**

# *PRESS Guideline* 2015— Search Submission & Peer Review Assessment

Reference: McGowan J, Sampson M, Salzwedel DM, Cogo E, Foerster V, Lefebvre C. PRESS Peer Review of Electronic Search Strategies: 2015 guideline statement. *J Clin Epidemiol* 2016;75:40-6. Available: <http://www.jclinepi.com/article/S0895-4356(16)00058-5/pdf>.

**Search submission: This section to be filled in by the searcher**

Searcher: Becky Skidmore Email: [bskidmore@rogers.com](mailto:bskidmore@rogers.com)

Date submitted: 29 Aug 2016 Date requested by: 2 Sep 2016

| 1. **Systematic Review Title** |  |
| --- | --- |

Systematic Literature Review and Network Meta-Analysis of Effectiveness Data for HERCEPTIN in HER2+ Early Breast Cancer

| 1. **This search strategy is …** |
| --- |

| x | My PRIMARY (core) database strategy — First time submitting a strategy for search question and database |
| --- | --- |
|  | My PRIMARY (core) strategy — Follow-up review NOT the first time submitting a strategy for search question and database. If this is a response to peer review, itemize the changes made to the review suggestions |
|  | SECONDARY search strategy— First time submitting a strategy for search question and database |
|  | SECONDARY search strategy — NOT the first time submitting a strategy for search question and database. If  this is a response to peer review, itemize the changes made to the review suggestions |

| 1. **Database** (e.g., MEDLINE, CINAHL) *[mandatory]* |
| --- |

MEDLINE

| 1. **Interface** (e.g., Ovid, EbscoHost…) *[mandatory]* |
| --- |

Ovid

| 1. **Research Question** (Describe the purpose of the search)  *[mandatory]* |
| --- |

| 1. **PICO Format** Outline the PICOs for your question — i.e., Patient, Intervention, Comparison, Outcome, and Study Design — as applicable |
| --- |

| **P** | HER2+ early breast cancer |
| --- | --- |
| **I** | HERCEPTIN in the early breast cancer setting (neoadjuvant and adjuvant) |
| **C** | \| CYCLOPHOSPHAMIDE: CYTOXAN \| \| --- \| \| DOXORUBICIN: RUBEX - ADRIAMYCIN \| \| EPIRUBICIN: ELLENCE \| \| 5-FLUOROURACIL: FLUOROPLEX - EFUDEX - ADRUCIL \| \| CAPECITABINE XELODA \| \| METHOTREXATE: TREXALL - METHOTREX - RHEUMATREX DP \| \| GEMCITABINE: GEMZAR \| \| CARBOPLATIN: PARAPLATIN \| \| CISPLATIN: PLATINOL \| \| DOCETAXEL: TAXOTERE \| \| PACLITAXEL: TAXOL \| \| PACLITAXEL - LIPOSOMAL: ABRAXANE \| \| VINORELBINE: NAVELBINE \| \| BEVACIZUMAB AVASTIN \| \| LAPATINIB TYKERB \| \| PERTUZUMAB PERJETA \| \| TRASTUZUMAB HERCEPTIN \| \| TRASTUZUMAB EMTANSINE: KADCYLA, T-DM1 \| \| NERATINIB \| \| ANASTROZOLE ARIMIDEX \| \| EXEMESTANE AROMASIN \| \| FULVESTRANT FASLODEX \| \| LETROZOLE FEMARA \| \| TAMOXIFEN: NOLVADEX- TAMOXIFEN CIT \| \| Any drug therapy for HER2+ adjuvant or neoadjuvant breast cancer \| |
| **O** | Not specified |
| **S** | Published studies and abstracts of randomized controlled trials (RCTs), comparative observational studies (e.g. case-control, cross-sectional, longitudinal, cohort studies), and meta-analyses; limit to humans |

| 1. **Inclusion Criteria** (List criteria such as age groups, study designs, etc., to be included) *[optional]*   **This search strategy is …** |
| --- |

As per PICO

| 1. **Exclusion Criteria** (List criteria such as study designs, date limits, etc., to be excluded) **[optional]** |
| --- |

Non-English (will confirm whether English to be removed at the search level or during screening**)**

| 1. **Was a search filter applied? Yes** |
| --- |

Amended Cochrane RCT filter plus filters largely based on CADTH’s range of filters

| 1. **Notes or comments you feel would be useful for the peer reviewer**  *[optional]* |
| --- |

Primary focus is to perform a systematic review on Herceptin. Comparators are included to assess feasibility of a network meta-analysis (confirmed in team discussions), which may be provided as part II of project.

Note: Had initially developed a strategy that created a separate concept for adjuvant/neoadjuvant ANDed with the drugs but am concerned that these terms will not always be mentioned in the context of the drugs. Have instead OR’d the concept of adjuvant chemotherapies with the names of the drugs identified by the research team as being of primary interest.

| 1. **Please copy and paste your search strategy here, exactly as run, including the number of hits per line. [mandatory]** |
| --- |

Database: Epub Ahead of Print, In-Process & Other Non-Indexed Citations, Ovid MEDLINE(R) Daily and Ovid MEDLINE(R) <1946 to Present>

Search Strategy:

--------------------------------------------------------------------------------

1 exp Breast Neoplasms/ (248365)

2 ((breast$1 or mamma or mammary) adj3 (adenocarcinoma* or cancer* or carcinoma* or neoplasm* or tumour* or tumor*)).tw,kw. (279494)

3 1 or 2 [BREAST CANCER] (336954)

4 Receptor, ErbB-2/ (19505)

5 (ErbB2 or "ErbB 2" or HER2* or "HER 2*" or "c-ErbB2" or "C-ErbB 2").tw,kw. (30985)

6 ((oncoprotein* or onco-protein* or protein* or receptor*) adj1 (neu or neuregulin)).tw,kw. (1211)

7 (CD340 adj1 antigen?).tw,kw. (0)

8 ("p185(c-neu)" or p185erbB).tw,kw. (54)

9 neu proto-oncogene protein*.tw,kw. (2)

10 metastatic lymph node gene 19 protein*.tw,kw. (0)

11 "luminal b".tw,kw. (1035)

12 luminal subtype*.tw,kw. (226)

13 (human adj1 "epidermal growth factor receptor 2").tw,kw. (4275)

14 Receptor, Epidermal Growth Factor/ (33552)

15 limit 14 to yr="2006-2007" (3066)

16 or/4-13,15 (37774)

17 3 and 16 [HER2 BREAST CANCER] (23764)

18 Combined Modality Therapy/ (154830)

19 limit 18 to yr="1984-1991" (33604)

20 Chemotherapy, Adjuvant/ (33779)

21 ((chemotherap* or chemo-therap*) adj5 (adjuvant* or adjuvent* or neoadjuvant* or neo-adjuvant* or neoadjuvent* or neo-adjuvent* or adjunct* or neoadjunct* or neo-adjunct*)).tw,kw. (35427)

22 ((drug therap* or pharmacothera* or pharmaco-therap*) adj5 (adjuvant* or adjuvent* or neoadjuvant* or neo-adjuvant* or neoadjuvent* or neo-adjuvent* or adjunct* or neoadjunct* or neo-adjunct*)).tw,kw. (763)

23 exp Breast Neoplasms/dt (45879)

24 exp Antineoplastic Agents/ (928335)

25 ("anti-HER2" or "anti-HER-2").tw,kw. (1866)

26 ((HER2 or "HER 2") adj1 block*).tw,kw. (163)

27 ((HER2 or "HER 2") adj1 antagoni*).tw,kw. (24)

28 Receptor, ErbB-2/ai [Antagonists & Inhibitors] (1807)

29 Antineoplastic Combined Chemotherapy Protocols/ (119184)

30 Trastuzumab/ (4956)

31 (herceptin* or trastuzumab* or TZM).tw,kw. (7981)

32 (kadcyla or pro 132365 or pro132365 or "t dm 1" or "t dm1" or tmab mcc dm1).tw,kw. (261)

33 trastuzumab.rn. (4956)

34 exp Cyclophosphamide/ (50198)

35 (alkyroxan or b 518 or b518 or carloxan or ciclofosfamida or ciclolen or ciclolen or cicloxal or clafen or cyclo-cell or cycloblastin or cycloblastine or cyclofosamide or cyclofosfamid or cyclofosfamide or cyclophar or cyclophosphamide or cyclophosphamide or cyclophosphamides or cyclophosphan or cyclophosphane or cyclostin or cycloxan or cyphos or cytophosphan or cytophosphane or cytoxan or endocyclophosphate or endoxan or endoxana or enduxan or genoxal or ledoxan or ledoxina or mitoxan or neosan or neosar or noristan or nsc 26271 or nsc26271 or nsc 2671 or nsc2671 or procytox or procytoxide or semdoxan or sendoxan or syklofosfamid).tw,kw. (44799)

36 cyclophosphamide.rn. (46691)

37 Doxorubicin/ (44378)

38 (adriablastin? or adriacin or adriamicin? or adriamycin? or adriblastin? or adrim or adrimedac or adrubicin or amminac or caelix or caelyx or carcinocin or "dox sl" or doxil or DOXO-cell or doxolem or "doxor lyo" or doxorubicin? or doxotec or evacet or farmiblastina or "fi 106" or fi106 or ifadox or lipodox or "mcc 465" or mcc465 or myocet* or nsc 123127 or nsc123127 or onkodox or rastocin? or resmycin or ribodoxo or rubex or rubidox or sarcodoxome or "tlc d 99").tw,kw. (47753)

39 doxorubicin.rn. (44378)

40 Epirubicin/ (4699)

41 (4'-Epi-Adriamycin or 4'-Epi-Doxorubicin or 4'-Epi-DXR or 4'-Epiadriamycin or 4'-Epidoxorubicin or binarin or ellence or EPI-cell or epiadriamycin or epidoxo or epidoxorubicin or epidx or epifil or epilem or epirubicin or farmorrubicina or farmorubicin? or IMI-28 or NSC-256942 or NSC256942 or pharmorubicin? or pidorubicin).tw,kw. (5327)

42 epirubicin.rn. (4699)

43 exp Fluorouracil/ (41991)

44 (5-FU or 5FU or 5-fluorouracil or 5fluorouracil or 5-fluoruracil or 5fluoruracil or accusite or actino-hermal or adrucil or carac or effluderm or efudex or efudix or efurix or f6627 or fivoflu or fluoro-uracile or fluoroblastin or fluoroplex or fluorouracil or fluoruracil or fluouracil or fluracedyl or flurodex or fluracil or fluracilium or fluril or fluroblastin or flurouracil or fluoxan or haemato-fu or ifacil or neofluor or nsc 18913 or nsc18913 or nsc 19893 or nsc19893 or oncofu or onkofluor or ribofluor or uflahex or utoral or verrumal).tw,kw. (38848)

45 Fluorouracil.rn. (38066)

46 Capecitabine/ (3418)

47 (apecitab or ecansya or capecitabine or xeloda).tw,kw. (4761)

48 capecitabine.rn. (3418)

49 methotrexate/ (34532)

50 (amethopterine or abitrexate or amethopterin or amethopterine or ametopterine or antifolan or biotrexate or canceren or cl 14377 or cl4377 or emtexate or emthexat or emthexate or emtrexate or enthexate or farmitrexat or farmitrexate or farmotrex or folex or ifamet or imeth or intradose MTX or lantarel or ledertrexate or maxtrex or metex or methoblastin or methohexate or methotrate or methotrexat or methotrexate or methotrexato or methoxtrexate or methrotrexate or methylaminopterin or methylaminopterine or meticil or metoject or metothrexate or metotrexat or metotrexate or metotrexin or metrex or mexate or mpi 5004 or mpi5004 or neotrexate or novatrex or nsc 740 or nsc740 or otrexup or rasuvo or reumatrex or rheumatrex or texate or texate-t or texorate or trexall or xaken or zexate).tw,kw. (36272)

51 methotrexate.rn. (34532)

52 gemcitabine.tw,kw. (12066)

53 (gemcitabine or gemcite or gemzar or ly 188011 or ly188011).tw,kw. (12093)

54 Carboplatin/ (10111)

55 (blastocarb or boplatex or carboplat or carboplatin or carboplatino or carbosin or carbotec or carplan or CBDCA or cycloplatin or erbakar or ercar or ifacap or kemocarb or nsc 241240 or nsc241240 or oncocarbin or paraplatin or paraplatine).tw,kw. (12488)

56 carboplatin.rn. (10111)

57 Cisplatin/ (45171)

58 (abiplatin or biocisplatinum or biocysplatinum or blastolem or briplatin or cis ddp or cis diamine dichloroplatinum or cis diaminechloroplatinum or cis diaminedichloroplatinum or cis diammine dichloroplatinum or cis diamminedichloroplatinum or cis dichloridiammineplatinum or cis dichloroadiamine platinum or cis dichlorodiamine platinum or cis dichlorodiamineplatinum or cis dichlorodiammine platinum or cis dichlorodiammineplatinum or cis platinous diamino dichloride or cis platinum or cisplatin or cisplatine or cisplatino or cisplatinum or cisplatyl or citoplatino or cytoplatin or cytosplat).tw,kw. (54443)

59 (diamine dichloroplatinum or diaminodichloroplatinum or diamminedichloroplatinum or dichlorodiamine platinum or dichlorodiammineplatinum or docistin or elvecis or kemoplat or lederplatin or lipoplatin or mpi 5010 or mpi5010 or neoplatin or niyaplat or nk 801 or nk801 or noveldexis or nsc 119875 or nsc119875 or platamine or platiblastin or platidiam or platimine or platinex or platinil or platinol or platinoxan or platiran or platistil or platistin or platosin or randa or romcis or sicatem or "spi 077" or tecnoplatin).tw,kw. (3172)

60 (platinum* adj1 (diaminodichloride or diamino dichloride or diamine dichloride or diaminedichloride or diaminodichloride or diamminedichloride)).tw,kw. (25)

61 cisplatin.rn. (45171)

62 docetaxel.tw,kw. (11515)

63 (daxotel or dexotel or docefrez or lit 976 or lit976 or nsc 628503 or nsc628503 or oncodocel or taxoter or taxotere or texot).tw,kw. (1136)

64 docetaxel.rn. (8478)

65 paclitaxel/ (22239)

66 ("abi 007" or abi007 or abraxane or anzatax or asotax or biotax or bms 181339 or bms181339 or bristaxol or britaxol or coroxane or formoxol or genexol or hunxol or ifaxol or infinnium or intaxel or "mbt 0206" or mbt0206 or medixel or mitotax or nsc 125973 or nsc125973 or oncogel or onxol or pacitaxel or pacxel or padexol or parexel or paxceed or paxene or paxus or praxel or taxocris or taxol or taxus or taycovit or yewtaxan).tw,kw. (8562)

67 paclitaxel.rn. (22239)

68 vinorelbine.tw,kw. (3130)

69 (anx 530 or anx530 or eunades or exelbine or kw 2307 or kw2307 or navelbin or navirel or vinbine or vinelbine or vinorelbine).tw,kw. (3136)

70 vinorelbine.rn. (2470)

71 Bevacizumab/ (8523)

72 (altuzan or avastin or bevacizumab or nsc 704865 or nsc704865).tw,kw. (11924)

73 bevacizumab.rn. (8523)

74 lapatinib.tw,kw. (1889)

75 (lapatinib or gw 2016 or gw2016 or gw 572016 or gw572016 or gw 572016f or gw572016f or tykerb or tyver).tw,kw. (1907)

76 pertuzumab.tw,kw. (506)

77 (monoclonal antibody 2C4 or omnitarg or perjeta or pertuzumab or rhumab 2C4).tw,kw. (519)

78 neratinib.tw,kw. (107)

79 (HKI 272 or HKI272 or way 177820 or way177820).tw,kw. (44)

80 anastrozole.tw,kw. (1504)

81 (arimidex or ici d1033 or icid1033 or trozolet or ZD-1033 or ZD1033).tw,kw. (258)

82 exemestane.tw,kw. (1044)

83 (aromasil or aromasin or aromasine or FCE 24304 or nikidess or pnu 155971 or pnu155971).tw,kw. (46)

84 fulvestrant.tw,kw. (963)

85 (faslodex or ICI 182,780 or ICI 182780 or zd 182780 or zd182780 or zd 9238 or zd9238 or zm 182780 or zm182780).tw,kw. (2536)

86 letrozole.tw,kw. (2129)

87 (CGS 20267 or CGS20267 or femar or femara).tw,kw. (139)

88 exp Tamoxifen/ (19574)

89 (ICI-46,474 or ICI-46474 or ICI-47699 or kessar or nolvadex or novaldex or nsc 180973 or nsc180973 or soltamox or tamoplac or tamoxasta or tamoxifen or tamoxifene or tomaxithen or zitazonium).tw,kw. (20290)

90 tamoxifen.rn. (17569)

91 or/19-90 [ADJUVANT/NEOADJUVANT CHEMOTHERAPY/THERAPIES, DRUGS OF INTEREST] (1079953)

92 17 and 91 [HER2 BREAST CANCER - ADJUVANT/NEOADJUVANT CHEMOTHERAPY/THERAPIES, DRUGS OF INTEREST] (11528)

93 exp Animals/ not (exp Animals/ and Humans/) (4303884)

94 92 not 93 [ANIMAL-ONLY REMOVED] (11305)

95 (comment or editorial or interview or news or newspaper article).pt. (1186922)

96 (letter not (letter and randomized controlled trial)).pt. (935716)

97 94 not (95 or 96) [OPINION PIECES REMOVED] (10723)

98 limit 97 to systematic reviews (364)

99 meta analysis.pt. (72737)

100 exp meta-analysis as topic/ (15322)

101 (meta-analy* or metanaly* or metaanaly* or met analy* or integrative research or integrative review* or integrative overview* or research integration or research overview* or collaborative review*).tw,kw. (105154)

102 (systematic review* or systematic overview* or evidence-based review* or evidence-based overview* or (evidence adj3 (review* or overview*)) or meta-review* or meta-overview* or meta-synthes* or rapid review* or "review of reviews" or technology assessment* or HTA or HTAs).tw,kw. (127318)

103 exp Technology assessment, biomedical/ (9798)

104 (cochrane or health technology assessment or evidence report).jw. (18250)

105 ((indirect* or mixed or multi-treatment*) adj2 compar*).tw,kw. (3551)

106 ((network* or network-based) adj (MA or MAs)).kw,tw. (3)

107 or/99-106 (232948)

108 97 and 107 (296)

109 98 or 108 [REVIEWS / META-ANALYSES] (465)

110 (controlled clinical trial or randomized controlled trial).pt. (515857)

111 "Clinical Trials as Topic".sh. (179120)

112 Randomized Controlled Trials as Topic/ (109469)

113 (randomi#ed or randomly or RCT$1 or placebo*).tw,kw. (763718)

114 ((singl* or doubl* or trebl* or tripl*) adj (mask* or blind* or dumm*)).tw,kw. (147534)

115 trial.ti. (160998)

116 or/110-115 (1151313)

117 97 and 116 [RCTS] (2027)

118 controlled clinical trial.pt. (91610)

119 Controlled Clinical Trial/ or Controlled Clinical Trials as Topic/ (96825)

120 (control* adj2 trial*).tw,kw. (193673)

121 Non-Randomized Controlled Trials as Topic/ (78)

122 (nonrandom* or non-random* or quasi-random* or quasi-experiment*).tw,kw. (42902)

123 (nRCT or nRCTs or non-RCT$1).tw,kw. (506)

124 Controlled Before-After Studies/ (182)

125 (control* adj3 ("before and after" or "before after")).tw,kw. (3354)

126 Interrupted Time Series Analysis/ (210)

127 (time series adj3 interrupt*).tw,kw. (1670)

128 (pre- adj3 post-).tw,kw. (56679)

129 (pretest adj3 posttest).tw,kw. (3863)

130 Historically Controlled Study/ (69)

131 (control* adj2 stud$3).tw,kw. (187462)

132 Control Groups/ (1605)

133 (control$ adj2 group$1).tw,kw. (390251)

134 trial.ti. (160998)

135 or/118-134 (952754)

136 97 and 135 [CCTS/NON-RCTS] (880)

137 exp Cohort Studies/ (1584556)

138 cohort?.tw,kw. (385543)

139 Retrospective Studies/ (601058)

140 (longitudinal or prospective or retrospective).tw,kw. (929551)

141 ((followup or follow-up) adj (study or studies)).tw,kw. (43490)

142 Observational study.pt. (25428)

143 (observation$2 adj (study or studies)).tw,kw. (66780)

144 ((population or population-based) adj (study or studies or analys#s)).tw,kw. (20558)

145 ((multidimensional or multi-dimensional) adj (study or studies)).tw,kw. (90)

146 Comparative Study.pt. (1765032)

147 ((comparative or comparison) adj (study or studies)).tw,kw. (91951)

148 exp Case-Control Studies/ (809227)

149 ((case-control* or case-based or case-comparison) adj (study or studies)).tw,kw. (83881)

150 Cross-Sectional Studies/ (225339)

151 ((crosssection* or cross-section*) adj (study or studies or survey?)).tw,kw. (125916)

152 or/137-151 (3923179)

153 97 and 152 [OBSERVATIONAL STUDIES] (2849)

154 109 or 117 or 136 or 153 [ALL STUDY DESIGNS] (4469)

***************************

**Peer review assessment: this section to be filled in by the reviewer**

|  | Reviewer: Kaitryn Campbell | Email: kaitryn_chris@sympatico.ca | Date completed: 30 Aug. 2016 |
| --- | --- | --- | --- |
|  |  |  |  |

Do you wish to be acknowledged? (If yes, the review team will be advised to add an acknowledgement to any publications related to this work.) Yes No

The suggested acknowledgement is “We thank Xxxxx Yyyyyy, MLIS, AHIP (xxxxx Health Sciences Library, University of xxxxxx) for peer review of the MEDLINE search strategy.” [please edit to indicate your name, postnomials and institutional affiliation as you would like them presented].

|  | **1. TRANSLATION** |  |  | | |
| --- | --- | --- | --- | --- | --- |
| A -­‐No revisions | | X |  |  |  |
| B -­‐ Revision(s) suggested | |  |  |  |  |
| C -­‐ Revision(s) required | |  |  |  |  |

If “B” or “C,” please provide an explanation or example:

**2. BOOLEAN AND PROXIMITY OPERATORS**

| A -­‐No revisions | X |
| --- | --- |
| B -­‐ Revision(s) suggested |  |
| C -­‐ Revision(s) required |  |

If “B” or “C,” please provide an explanation or example:

**3. SUBJECT HEADINGS**

| A -­‐No revisions |  |
| --- | --- |
| B -­‐ Revision(s) suggested | X |
| C -­‐ Revision(s) required |  |

If “B” or “C,” please provide an explanation or example:

For HER2+ concept, consider adding: ERBB2 protein, human.nm.

**4. TEXT WORD SEARCHING**

| A -­‐No revisions |  |
| --- | --- |
| B -­‐ Revision(s)suggested | X |
| C -­‐ Revision(s) required |  |

If “B” or “C,” please provide an explanation or example:

Line 35, consider deleting duplicates (2 crossed out in following): (alkyroxan or b 518 or b518 or carloxan or ciclofosfamida or ciclolen or ~~ciclolen~~ or cicloxal or clafen or cyclo-cell or cycloblastin or cycloblastine or cyclofosamide or cyclofosfamid or cyclofosfamide or cyclophar or cyclophosphamide or ~~cyclophosphamide~~ or cyclophosphamides or cyclophosphan or cyclophosphane or cyclostin or cycloxan or cyphos or cytophosphan or cytophosphane or cytoxan or endocyclophosphate or endoxan or endoxana or enduxan or genoxal or ledoxan or ledoxina or mitoxan or neosan or neosar or noristan or nsc 26271 or nsc26271 or nsc 2671 or nsc2671 or procytox or procytoxide or semdoxan or sendoxan or syklofosfamid).tw,kw.

**5. SPELLING, SYNTAX, AND LINE NUMBERS**

| A -­‐No revisions | X |
| --- | --- |
| B -­‐ Revision(s)suggested |  |
| C -­‐ Revision(s) required |  |

If “B” or “C,” please provide an explanation or example:

**6. LIMITS AND FILTERS**

| A -­‐No revisions | X |
| --- | --- |
| B -­‐ Revision(s) suggested |  |
| C -­‐ Revision(s) required |  |

If “B” or “C,” please provide an explanation or example:

OVERALL EVALUATION (Note: If one or more “revision required” is noted above, the response below must be “revisions required”.)

| A -­‐No revisions |  |
| --- | --- |
| B -­‐ Revision(s) suggested | X |
| C -­‐ Revision(s) required |  |

Additional comments:
