# Supplementary material for: Herceptin® (trastuzumab) in HER2-positive early breast cancer: protocol for a systematic review and cumulative network meta-analysis
Source: Syst Rev. 2017 Oct 10;6:196. doi: 10.1186/s13643-017-0588-2 (PMC5634826; doi:10.1186/s13643-017-0588-2)
Supplement: Supplementary file 3 — Search Strategy. Contains the search strategy to be used for the planned systematic literature review. (DOCX 37 kb) [file 13643_2017_588_MOESM3_ESM.docx]

**Additional file 3: Search Strategy**

HER2 Breast Cancer

Final Strategies – OVID, Cochrane

2016 Oct 19

OVID Multifile

Database: Embase <1988 to 2016 Week 42>, Epub Ahead of Print, In-Process & Other Non-Indexed Citations, Ovid MEDLINE(R) Daily and Ovid MEDLINE(R) <1946 to Present>

Search Strategy:

--------------------------------------------------------------------------------

1 exp Breast Neoplasms/ (647870)

2 ((breast$1 or mamma or mammary) adj3 (adenocarcinoma* or cancer* or carcinoma* or neoplasm* or tumour* or tumor*)).tw,kw. (624530)

3 1 or 2 [BREAST CANCER] (774147)

4 Receptor, ErbB-2/ (51944)

5 ERBB2 protein, human.nm. (6540)

6 (ErbB2 or "ErbB 2" or HER2* or "HER 2*" or "c-ErbB2" or "C-ErbB 2").tw,kw. (84076)

7 ((oncoprotein* or onco-protein* or protein* or receptor*) adj1 (neu or neuregulin)).tw,kw. (2743)

8 (CD340 adj1 antigen?).tw,kw. (0)

9 ("p185(c-neu)" or p185erbB).tw,kw. (95)

10 neu proto-oncogene protein*.tw,kw. (4)

11 metastatic lymph node gene 19 protein*.tw,kw. (0)

12 "luminal b".tw,kw. (3500)

13 luminal subtype*.tw,kw. (711)

14 (human adj1 "epidermal growth factor receptor 2").tw,kw. (10097)

15 Receptor, Epidermal Growth Factor/ (82682)

16 limit 15 to yr="2006-2007" (7236)

17 or/4-14,16 (106066)

18 3 and 17 [HER2 BREAST CANCER] (70383)

19 Combined Modality Therapy/ (202482)

20 limit 19 to yr="1984-1991" (40042)

21 Chemotherapy, Adjuvant/ (74848)

22 ((chemotherap* or chemo-therap*) adj5 (adjuvant* or adjuvent* or neoadjuvant* or neo-adjuvant* or neoadjuvent* or neo-adjuvent* or adjunct* or neoadjunct* or neo-adjunct*)).tw,kw. (90712)

23 ((drug therap* or pharmacothera* or pharmaco-therap*) adj5 (adjuvant* or adjuvent* or neoadjuvant* or neo-adjuvant* or neoadjuvent* or neo-adjuvent* or adjunct* or neoadjunct* or neo-adjunct*)).tw,kw. (1733)

24 exp Breast Neoplasms/dt (126897)

25 exp Antineoplastic Agents/ (2507071)

26 ("anti-HER2" or "anti-HER-2").tw,kw. (5057)

27 ((HER2 or "HER 2") adj1 block*).tw,kw. (471)

28 ((HER2 or "HER 2") adj1 antagoni*).tw,kw. (65)

29 Receptor, ErbB-2/ai [Antagonists & Inhibitors] (1848)

30 Antineoplastic Combined Chemotherapy Protocols/ (313774)

31 Trastuzumab/ (34858)

32 (herceptin* or trastuzumab* or TZM).tw,kw. (27821)

33 (ado-trastuzumab* or kadcyla or pro 132365 or pro132365 or "t dm 1" or "t dm1" or tmab mcc dm1).tw,kw. (1200)

34 trastuzumab.rn. (28831)

35 exp Cyclophosphamide/ (205396)

36 (alkyroxan or b 518 or b518 or carloxan or ciclofosfamida or ciclolen or cicloxal or clafen or cyclo-cell or cycloblastin or cycloblastine or cyclofosamide or cyclofosfamid or cyclofosfamide or cyclophar or cyclophosphamide or cyclophosphamides or cyclophosphan or cyclophosphane or cyclostin or cycloxan or cyphos or cytophosphan or cytophosphane or cytoxan or endocyclophosphate or endoxan or endoxana or enduxan or genoxal or ledoxan or ledoxina or mitoxan or neosan or neosar or noristan or nsc 26271 or nsc26271 or nsc 2671 or nsc2671 or procytox or procytoxide or semdoxan or sendoxan or syklofosfamid).tw,kw. (97880)

37 cyclophosphamide.rn. (187679)

38 Doxorubicin/ (182708)

39 (adriablastin? or adriacin or adriamicin? or adriamycin? or adriblastin? or adrim or adrimedac or adrubicin or amminac or caelix or caelyx or carcinocin or "dox sl" or doxil or DOXO-cell or doxolem or "doxor lyo" or doxorubicin? or doxotec or evacet or farmiblastina or "fi 106" or fi106 or ifadox or lipodox or "mcc 465" or mcc465 or myocet? or nsc 123127 or nsc123127 or onkodox or rastocin? or resmycin or ribodoxo or rubex or rubidox or sarcodoxome or "tlc d 99").tw,kw. (109652)

40 doxorubicin.rn. (171278)

41 Epirubicin/ (29051)

42 (4'-Epi-Adriamycin or 4'-Epi-Doxorubicin or 4'-Epi-DXR or 4'-Epiadriamycin or 4'-Epidoxorubicin or binarin or ellence or EPI-cell or epiadriamycin or epidoxo or epidoxorubicin or epidx or epifil or epilem or epirubicin or farmorrubicina or farmorubicin? or IMI-28 or NSC-256942 or NSC256942 or pharmorubicin? or pidorubicin).tw,kw. (13010)

43 epirubicin.rn. (27584)

44 exp Fluorouracil/ (145927)

45 (5-FU or 5FU or 5-fluorouracil or 5fluorouracil or 5-fluoruracil or 5fluoruracil or accusite or actino-hermal or adrucil or carac or effluderm or efudex or efudix or efurix or f6627 or fivoflu or fluoro-uracile or fluoroblastin or fluoroplex or fluorouracil or fluoruracil or fluouracil or fluracedyl or flurodex or fluracil or fluracilium or fluril or fluroblastin or flurouracil or fluoxan or haemato-fu or ifacil or neofluor or nsc 18913 or nsc18913 or nsc 19893 or nsc19893 or oncofu or onkofluor or ribofluor or uflahex or utoral or verrumal).tw,kw. (85471)

46 Fluorouracil.rn. (132472)

47 Capecitabine/ (25508)

48 (apecitab or ecansya or capecitabine or xeloda).tw,kw. (15308)

49 capecitabine.rn. (21653)

50 methotrexate/ (162708)

51 (amethopterine or abitrexate or amethopterin or amethopterine or ametopterine or antifolan or biotrexate or canceren or cl 14377 or cl4377 or emtexate or emthexat or emthexate or emtrexate or enthexate or farmitrexat or farmitrexate or farmotrex or folex or ifamet or imeth or intradose MTX or lantarel or ledertrexate or maxtrex or metex or methoblastin or methohexate or methotrate or methotrexat or methotrexate or methotrexato or methoxtrexate or methrotrexate or methylaminopterin or methylaminopterine or meticil or metoject or metothrexate or metotrexat or metotrexate or metotrexin or metrex or mexate or mpi 5004 or mpi5004 or neotrexate or novatrex or nsc 740 or nsc740 or otrexup or rasuvo or reumatrex or rheumatrex or texate or texate-t or texorate or trexall or xaken or zexate).tw,kw. (82437)

52 methotrexate.rn. (146922)

53 gemcitabine.tw,kw. (32864)

54 (gemcite or gemzar or ly 188011 or ly188011).tw,kw. (2207)

55 Carboplatin/ (64243)

56 (blastocarb or boplatex or carboplat or carboplatin or carboplatino or carbosin or carbotec or carplan or CBDCA or cycloplatin or erbakar or ercar or ifacap or kemocarb or nsc 241240 or nsc241240 or oncocarbin or paraplatin or paraplatine).tw,kw. (33222)

57 carboplatin.rn. (58198)

58 Cisplatin/ (185363)

59 (abiplatin or biocisplatinum or biocysplatinum or blastolem or briplatin or cis ddp or cis diamine dichloroplatinum or cis diaminechloroplatinum or cis diaminedichloroplatinum or cis diammine dichloroplatinum or cis diamminedichloroplatinum or cis dichloridiammineplatinum or cis dichloroadiamine platinum or cis dichlorodiamine platinum or cis dichlorodiamineplatinum or cis dichlorodiammine platinum or cis dichlorodiammineplatinum or cis platinous diamino dichloride or cis platinum or cisplatin or cisplatine or cisplatino or cisplatinum or cisplatyl or citoplatino or cytoplatin or cytosplat).tw,kw. (124715)

60 (diamine dichloroplatinum or diaminodichloroplatinum or diamminedichloroplatinum or dichlorodiamine platinum or dichlorodiammineplatinum or docistin or elvecis or kemoplat or lederplatin or lipoplatin or mpi 5010 or mpi5010 or neoplatin or niyaplat or nk 801 or nk801 or noveldexis or nsc 119875 or nsc119875 or platamine or platiblastin or platidiam or platimine or platinex or platinil or platinol or platinoxan or platiran or platistil or platistin or platosin or randa or romcis or sicatem or "spi 077" or tecnoplatin).tw,kw. (6592)

61 (platinum* adj1 (diaminodichloride or diamino dichloride or diamine dichloride or diaminedichloride or diaminodichloride or diamminedichloride)).tw,kw. (31)

62 cisplatin.rn. (169431)

63 docetaxel.tw,kw. (32095)

64 (daxotel or dexotel or docefrez or lit 976 or lit976 or nsc 628503 or nsc628503 or oncodocel or taxoter or taxotere or texot).tw,kw. (5319)

65 docetaxel.rn. (46280)

66 paclitaxel/ (105768)

67 ("abi 007" or abi007 or abraxane or anzatax or asotax or biotax or bms 181339 or bms181339 or bristaxol or britaxol or coroxane or formoxol or genexol or hunxol or ifaxol or infinnium or intaxel or "mbt 0206" or mbt0206 or medixel or mitotax or nsc 125973 or nsc125973 or oncogel or onxol or pacitaxel or pacxel or padexol or parexel or paxceed or paxene or paxus or praxel or taxocris or taxol or taxus or taycovit or yewtaxan).tw,kw. (25296)

68 paclitaxel.rn. (94408)

69 vinorelbine.tw,kw. (8319)

70 (anx 530 or anx530 or eunades or exelbine or kw 2307 or kw2307 or navelbin or navelbine or navirel or vinbine or vinelbine).tw,kw. (1467)

71 vinorelbine.rn. (2588)

72 Bevacizumab/ (51774)

73 (altuzan or avastin or bevacizumab or nsc 704865 or nsc704865).tw,kw. (38470)

74 bevacizumab.rn. (43545)

75 lapatinib.tw,kw. (5770)

76 (gw 2016 or gw2016 or gw 572016 or gw572016 or gw 572016f or gw572016f or tykerb or tyver).tw,kw. (1782)

77 pertuzumab.tw,kw. (1594)

78 (monoclonal antibody 2C4 or omnitarg or perjeta or rhumab 2C4).tw,kw. (513)

79 neratinib.tw,kw. (408)

80 (HKI 272 or HKI272 or way 177820 or way177820).tw,kw. (467)

81 anastrozole.tw,kw. (4015)

82 (arimidex or ici d1033 or icid1033 or trozolet or ZD-1033 or ZD1033).tw,kw. (1935)

83 exemestane.tw,kw. (3002)

84 (aromasil or aromasin or aromasine or FCE 24304 or nikidess or pnu 155971 or pnu155971).tw,kw. (617)

85 fulvestrant.tw,kw. (2709)

86 (faslodex or ICI 182,780 or ICI 182780 or zd 182780 or zd182780 or zd 9238 or zd9238 or zm 182780 or zm182780).tw,kw. (6794)

87 letrozole.tw,kw. (5868)

88 (CGS 20267 or CGS20267 or femar or femara).tw,kw. (1330)

89 exp Tamoxifen/ (70239)

90 (ICI-46,474 or ICI-46474 or ICI-47699 or kessar or nolvadex or novaldex or nsc 180973 or nsc180973 or soltamox or tamoplac or tamoxasta or tamoxifene or tomaxithen or zitazonium).tw,kw. (1611)

91 tamoxifen.rn. (61828)

92 or/20-91 [ADJUVANT/NEOADJUVANT CHEMOTHERAPY/THERAPIES, DRUGS OF INTEREST] (2702685)

93 18 and 92 [HER2 BREAST CANCER - ADJUVANT/NEOADJUVANT CHEMOTHERAPY/THERAPIES, DRUGS OF INTEREST] (38706)

94 exp Animals/ not (exp Animals/ and Humans/) (13839772)

95 93 not 94 [ANIMAL-ONLY REMOVED] (21398)

96 (comment or editorial or interview or news or newspaper article).pt. (1675306)

97 (letter not (letter and randomized controlled trial)).pt. (1767472)

98 95 not (96 or 97) [OPINION PIECES REMOVED] (20328)

99 limit 98 to yr="1990-current" [DATE LIMITS APPLIED] (20317)

100 limit 99 to english language [LIMITED TO ENGLISH LANGUAGE] (18949)

101 limit 100 to systematic reviews [Limit not valid in Embase; records were retained] (9238)

102 meta analysis.pt. (74283)

103 exp meta-analysis as topic/ (50700)

104 (meta-analy* or metanaly* or metaanaly* or met analy* or integrative research or integrative review* or integrative overview* or research integration or research overview* or collaborative review*).tw,kw. (244505)

105 (systematic review* or systematic overview* or evidence-based review* or evidence-based overview* or (evidence adj3 (review* or overview*)) or meta-review* or meta-overview* or meta-synthes* or rapid review* or "review of reviews" or technology assessment* or HTA or HTAs).tw,kw. (284824)

106 exp Technology assessment, biomedical/ (20916)

107 (cochrane or health technology assessment or evidence report).jw. (34688)

108 ((indirect* or mixed or multi-treatment*) adj2 compar*).tw,kw. (8737)

109 ((network* or network-based) adj (MA or MAs)).kw,tw. (11)

110 or/102-109 (523989)

111 100 and 110 (450)

112 101 or 111 [REVIEWS / META-ANALYSES] (9333)

113 (controlled clinical trial or randomized controlled trial).pt. (519865)

114 "Clinical Trials as Topic".sh. (180217)

115 Randomized Controlled Trials as Topic/ (166815)

116 (randomi#ed or randomly or RCT$1 or placebo*).tw,kw. (1750023)

117 ((singl* or doubl* or trebl* or tripl*) adj (mask* or blind* or dumm*)).tw,kw. (318239)

118 trial.ti. (356638)

119 or/113-118 (2235549)

120 100 and 119 [RCTS] (3224)

121 controlled clinical trial.pt. (91818)

122 Controlled Clinical Trial/ or Controlled Clinical Trials as Topic/ (545895)

123 (control* adj2 trial*).tw,kw. (439962)

124 Non-Randomized Controlled Trials as Topic/ (10328)

125 (nonrandom* or non-random* or quasi-random* or quasi-experiment*).tw,kw. (91512)

126 (nRCT or nRCTs or non-RCT$1).tw,kw. (1197)

127 Controlled Before-After Studies/ (168702)

128 (control* adj3 ("before and after" or "before after")).tw,kw. (7254)

129 Interrupted Time Series Analysis/ (152748)

130 (time series adj3 interrupt*).tw,kw. (3573)

131 (pre- adj3 post-).tw,kw. (148690)

132 (pretest adj3 posttest).tw,kw. (7872)

133 Historically Controlled Study/ (187915)

134 (control* adj2 stud$3).tw,kw. (417335)

135 Control Groups/ (258423)

136 (control$ adj2 group$1).tw,kw. (900863)

137 trial.ti. (356638)

138 or/121-137 (2546593)

139 100 and 138 [CCTS/NON-RCTS] (1953)

140 exp Cohort Studies/ (1894487)

141 cohort?.tw,kw. (996615)

142 Retrospective Studies/ (842418)

143 (longitudinal or prospective or retrospective).tw,kw. (2225517)

144 ((followup or follow-up) adj (study or studies)).tw,kw. (91372)

145 Observational study.pt. (26943)

146 (observation$2 adj (study or studies)).tw,kw. (169328)

147 ((population or population-based) adj (study or studies or analys#s)).tw,kw. (38443)

148 ((multidimensional or multi-dimensional) adj (study or studies)).tw,kw. (185)

149 Comparative Study.pt. (1771349)

150 ((comparative or comparison) adj (study or studies)).tw,kw. (179471)

151 exp Case-Control Studies/ (955328)

152 ((case-control* or case-based or case-comparison) adj (study or studies)).tw,kw. (186400)

153 Cross-Sectional Studies/ (285889)

154 ((crosssection* or cross-section*) adj (study or studies or survey?)).tw,kw. (288696)

155 or/140-154 (6162140)

156 100 and 155 [OBSERVATIONAL STUDIES] (4229)

157 112 or 120 or 139 or 156 [ALL STUDY DESIGNS] (13191)

158 157 use ppez (4307) [MEDLINE RECORDS]

159 exp breast cancer/ (603759)

160 ((breast$1 or mamma or mammary) adj3 (adenocarcinoma* or cancer* or carcinoma* or neoplasm* or tumour* or tumor*)).tw,kw. (624530)

161 159 or 160 [BREAST CANCER] (759845)

162 epidermal growth factor receptor 2/ (35048)

163 (ErbB2 or "ErbB 2" or HER2* or "HER 2*" or "c-ErbB2" or "C-ErbB 2").tw,kw. (84076)

164 ((oncoprotein* or onco-protein* or protein* or receptor*) adj1 (neu or neuregulin)).tw,kw. (2743)

165 (CD340 adj1 antigen?).tw,kw. (0)

166 ("p185(c-neu)" or p185erbB).tw,kw. (95)

167 neu proto-oncogene protein*.tw,kw. (4)

168 metastatic lymph node gene 19 protein*.tw,kw. (0)

169 "luminal b".tw,kw. (3500)

170 luminal subtype*.tw,kw. (711)

171 (human adj1 "epidermal growth factor receptor 2").tw,kw. (10097)

172 or/162-171 (97938)

173 161 and 172 [HER2 BREAST CANCER] (68384)

174 adjuvant chemotherapy/ (79476)

175 ((chemotherap* or chemo-therap*) adj5 (adjuvant* or adjuvent* or neoadjuvant* or neo-adjuvant* or neoadjuvent* or neo-adjuvent* or adjunct* or neoadjunct* or neo-adjunct*)).tw,kw. (90712)

176 ((drug therap* or pharmacothera* or pharmaco-therap*) adj5 (adjuvant* or adjuvent* or neoadjuvant* or neo-adjuvant* or neoadjuvent* or neo-adjuvent* or adjunct* or neoadjunct* or neo-adjunct*)).tw,kw. (1733)

177 exp breast cancer/dt (120515)

178 exp antineoplastic agent/ (1572365)

179 ("anti-HER2" or "anti-HER-2").tw,kw. (5057)

180 ((HER2 or "HER 2") adj1 block*).tw,kw. (471)

181 ((HER2 or "HER 2") adj1 antagoni*).tw,kw. (65)

182 exp cancer chemotherapy/ (269320)

183 trastuzumab/ (34858)

184 trastuzumab emtansine/ (1403)

185 (herceptin* or trastuzumab* or TZM).tw,kw. (27821)

186 (ado-trastuzumab* or kadcyla or pro 132365 or pro132365 or "t dm 1" or "t dm1" or tmab mcc dm1).tw,kw. (1200)

187 trastuzumab.rn. (28831)

188 trastuzumab emtansine.rn. (1059)

189 exp cyclophosphamide/ (205396)

190 (alkyroxan or b 518 or b518 or carloxan or ciclofosfamida or ciclolen or cicloxal or clafen or cyclo-cell or cycloblastin or cycloblastine or cyclofosamide or cyclofosfamid or cyclofosfamide or cyclophar or cyclophosphamide or cyclophosphamides or cyclophosphan or cyclophosphane or cyclostin or cycloxan or cyphos or cytophosphan or cytophosphane or cytoxan or endocyclophosphate or endoxan or endoxana or enduxan or genoxal or ledoxan or ledoxina or mitoxan or neosan or neosar or noristan or nsc 26271 or nsc26271 or nsc 2671 or nsc2671 or procytox or procytoxide or semdoxan or sendoxan or syklofosfamid).tw,kw. (97880)

191 cyclophosphamide.rn. (187679)

192 doxorubicin/ (182708)

193 (adriablastin? or adriacin or adriamicin? or adriamycin? or adriblastin? or adrim or adrimedac or adrubicin or amminac or caelix or caelyx or carcinocin or "dox sl" or doxil or DOXO-cell or doxolem or "doxor lyo" or doxorubicin? or doxotec or evacet or farmiblastina or "fi 106" or fi106 or ifadox or lipodox or "mcc 465" or mcc465 or myocet* or nsc 123127 or nsc123127 or onkodox or rastocin? or resmycin or ribodoxo or rubex or rubidox or sarcodoxome or "tlc d 99").tw,kw. (109657)

194 doxorubicin.rn. (171278)

195 epirubicin/ (29051)

196 (4'-Epi-Adriamycin or 4'-Epi-Doxorubicin or 4'-Epi-DXR or 4'-Epiadriamycin or 4'-Epidoxorubicin or binarin or ellence or EPI-cell or epiadriamycin or epidoxo or epidoxorubicin or epidx or epifil or epilem or epirubicin or farmorrubicina or farmorubicin? or IMI-28 or NSC-256942 or NSC256942 or pharmorubicin? or pidorubicin).tw,kw. (13010)

197 epirubicin.rn. (27584)

198 exp fluorouracil/ (145927)

199 (5-FU or 5FU or 5-fluorouracil or 5fluorouracil or 5-fluoruracil or 5fluoruracil or accusite or actino-hermal or adrucil or carac or effluderm or efudex or efudix or efurix or f6627 or fivoflu or fluoro-uracile or fluoroblastin or fluoroplex or fluorouracil or fluoruracil or fluouracil or fluracedyl or flurodex or fluracil or fluracilium or fluril or fluroblastin or flurouracil or fluoxan or haemato-fu or ifacil or neofluor or nsc 18913 or nsc18913 or nsc 19893 or nsc19893 or oncofu or onkofluor or ribofluor or uflahex or utoral or verrumal).tw,kw. (85471)

200 fluorouracil.rn. (132472)

201 capecitabine/ (25508)

202 (apecitab or ecansya or capecitabine or xeloda).tw,kw. (15308)

203 capecitabine.rn. (21653)

204 methotrexate/ (162708)

205 (amethopterine or abitrexate or amethopterin or amethopterine or ametopterine or antifolan or biotrexate or canceren or cl 14377 or cl4377 or emtexate or emthexat or emthexate or emtrexate or enthexate or farmitrexat or farmitrexate or farmotrex or folex or ifamet or imeth or intradose MTX or lantarel or ledertrexate or maxtrex or metex or methoblastin or methohexate or methotrate or methotrexat or methotrexate or methotrexato or methoxtrexate or methrotrexate or methylaminopterin or methylaminopterine or meticil or metoject or metothrexate or metotrexat or metotrexate or metotrexin or metrex or mexate or mpi 5004 or mpi5004 or neotrexate or novatrex or nsc 740 or nsc740 or otrexup or rasuvo or reumatrex or rheumatrex or texate or texate-t or texorate or trexall or xaken or zexate).tw,kw. (82437)

206 methotrexate.rn. (146922)

207 gemcitabine/ (43132)

208 (gemcitabine or gemcite or gemzar or ly 188011 or ly188011).tw,kw. (33793)

209 gemcitabine.rn. (44783)

210 carboplatin/ (64243)

211 (blastocarb or boplatex or carboplat or carboplatin or carboplatino or carbosin or carbotec or carplan or CBDCA or cycloplatin or erbakar or ercar or ifacap or kemocarb or nsc 241240 or nsc241240 or oncocarbin or paraplatin or paraplatine).tw,kw. (33222)

212 carboplatin.rn. (58198)

213 cisplatin/ (185363)

214 (abiplatin or biocisplatinum or biocysplatinum or blastolem or briplatin or cis ddp or cis diamine dichloroplatinum or cis diaminechloroplatinum or cis diaminedichloroplatinum or cis diammine dichloroplatinum or cis diamminedichloroplatinum or cis dichloridiammineplatinum or cis dichloroadiamine platinum or cis dichlorodiamine platinum or cis dichlorodiamineplatinum or cis dichlorodiammine platinum or cis dichlorodiammineplatinum or cis platinous diamino dichloride or cis platinum or cisplatin or cisplatine or cisplatino or cisplatinum or cisplatyl or citoplatino or cytoplatin or cytosplat).tw,kw. (124715)

215 (diamine dichloroplatinum or diaminodichloroplatinum or diamminedichloroplatinum or dichlorodiamine platinum or dichlorodiammineplatinum or docistin or elvecis or kemoplat or lederplatin or lipoplatin or mpi 5010 or mpi5010 or neoplatin or niyaplat or nk 801 or nk801 or noveldexis or nsc 119875 or nsc119875 or platamine or platiblastin or platidiam or platimine or platinex or platinil or platinol or platinoxan or platiran or platistil or platistin or platosin or randa or romcis or sicatem or "spi 077" or tecnoplatin).tw,kw. (6592)

216 (platinum* adj1 (diaminodichloride or diamino dichloride or diamine dichloride or diaminedichloride or diaminodichloride or diamminedichloride)).tw,kw. (31)

217 cisplatin.rn. (169431)

218 docetaxel/ (45177)

219 (daxotel or dexotel or docefrez or docetaxel or lit 976 or lit976 or nsc 628503 or nsc628503 or oncodocel or taxoter or taxotere or texot).tw,kw. (34659)

220 docetaxel.rn. (46280)

221 paclitaxel/ (105768)

222 ("abi 007" or abi007 or abraxane or anzatax or asotax or biotax or bms 181339 or bms181339 or bristaxol or britaxol or coroxane or formoxol or genexol or hunxol or ifaxol or infinnium or intaxel or "mbt 0206" or mbt0206 or medixel or mitotax or nsc 125973 or nsc125973 or oncogel or onxol or pacitaxel or pacxel or padexol or parexel or paxceed or paxene or paxus or praxel or taxocris or taxol or taxus or taycovit or yewtaxan).tw,kw. (25296)

223 paclitaxel.rn. (94408)

224 navelbine/ (15358)

225 (anx 530 or anx530 or eunades or exelbine or kw 2307 or kw2307 or navelbin or navelbine or navirel or vinbine or vinelbine or vinorelbine).tw,kw. (9106)

226 vinorelbine.rn. (2588)

227 bevacizumab/ (51774)

228 (altuzan or avastin or bevacizumab or nsc 704865 or nsc704865).tw,kw. (38470)

229 bevacizumab.rn. (43545)

230 lapatinib/ (9527)

231 (lapatinib or gw 2016 or gw2016 or gw 572016 or gw572016 or gw 572016f or gw572016f or tykerb or tyver).tw,kw. (7022)

232 lapatinib.rn. (9077)

233 pertuzumab/ (2858)

234 (monoclonal antibody 2C4 or omnitarg or perjeta or pertuzumab or rhumab 2C4).tw,kw. (1937)

235 pertuzumab.rn. (312)

236 neratinib/ (850)

237 (HKI 272 or HKI272 or neratinib or way 177820 or way177820).tw,kw. (793)

238 neratinib.rn. (719)

239 anastrozole/ (8072)

240 (anastrozole or arimidex or ici d1033 or icid1033 or trozolet or ZD-1033 or ZD1033).tw,kw. (5187)

241 anastrozole.rn. (8477)

242 exemestane/ (5039)

243 (aromasil or aromasin or aromasine or exemestane or FCE 24304 or nikidess or pnu 155971 or pnu155971).tw,kw. (3406)

244 exemestane.rn. (5007)

245 fulvestrant/ (6707)

246 (faslodex or fulvestrant or ICI 182,780 or ICI 182780 or zd 182780 or zd182780 or zd 9238 or zd9238 or zm 182780 or zm182780).tw,kw. (8906)

247 fulvestrant.rn. (7881)

248 letrozole/ (9068)

249 (CGS 20267 or CGS20267 or femar or femara or letrozole).tw,kw. (6627)

250 letrozole.rn. (9393)

251 exp Tamoxifen/ (70239)

252 (ICI-46,474 or ICI-46474 or ICI-47699 or kessar or nolvadex or novaldex or nsc 180973 or nsc180973 or soltamox or tamoplac or tamoxasta or tamoxifene or tomaxithen or zitazonium).tw,kw. (1611)

253 tamoxifen.rn. (61828)

254 or/174-253 [ADJUVANT/NEOADJUVANT CHEMOTHERAPY/THERAPIES, DRUGS OF INTEREST] (2029198)

255 173 and 254 [HER2 BREAST CANCER - ADJUVANT/NEOADJUVANT CHEMOTHERAPY/THERAPIES, DRUGS OF INTEREST] (37541)

256 exp animal experimentation/ or exp models animal/ or exp animal experiment/ or nonhuman/ or exp vertebrate/ (39205011)

257 exp human/ or exp human experimentation/ or exp human experiment/ (31545349)

258 256 not 257 (7660817)

259 255 not 258 [ANIMAL-ONLY REMOVED] (36735)

260 editorial.pt. (909450)

261 letter.pt. not (letter.pt. and randomized controlled trial/) (1762470)

262 259 not (260 or 261) [OPINION PIECES REMOVED] (35567)

263 limit 262 to yr="1990-current" [DATE LIMITS APPLIED] (35528)

264 limit 263 to english language [LIMITED TO ENGLISH LANGUAGE] (33570)

265 meta-analysis/ (223804)

266 "systematic review"/ (141277)

267 "meta analysis (topic)"/ (35236)

268 (meta-analy* or metanaly* or metaanaly* or met analy* or integrative research or integrative review* or integrative overview* or research integration or research overview* or collaborative review*).tw,kw. (244505)

269 (systematic review* or systematic overview* or evidence-based review* or evidence-based overview* or (evidence adj3 (review* or overview*)) or meta-review* or meta-overview* or meta-synthes* or rapid review* or "review of reviews" or technology assessment* or HTA or HTAs).tw,kw. (284824)

270 biomedical technology assessment/ (19807)

271 (cochrane or health technology assessment or evidence report).jw. (34688)

272 ((indirect* or mixed or multi-treatment*) adj2 compar*).tw,kw. (8737)

273 ((network* or network-based) adj (MA or MAs)).kw,tw. (11)

274 or/265-273 (566686)

275 264 and 274 [REVIEWS / META-ANALYSES] (1057)

276 randomized controlled trial/ or controlled clinical trial/ (1116359)

277 exp "clinical trial (topic)"/ (260844)

278 (randomi#ed or randomly or RCT$1 or placebo*).tw,kw. (1750023)

279 ((singl* or doubl* or trebl* or tripl*) adj (mask* or blind* or dumm*)).tw,kw. (318239)

280 trial.ti. (356638)

281 or/276-280 (2419104)

282 264 and 281 [RCTS] (7298)

283 exp controlled clinical trial/ (1116483)

284 exp "controlled clinical trial (topic)"/ (127869)

285 (control* adj2 trial*).tw,kw. (439962)

286 (nonrandom* or non-random* or quasi-random* or quasi-experiment*).tw,kw. (91512)

287 (nRCT or nRCTs or non-RCT$1).tw,kw. (1197)

288 (control* adj3 ("before and after" or "before after")).tw,kw. (7254)

289 time series analysis/ (23327)

290 (time series adj3 interrupt*).tw,kw. (3573)

291 pretest posttest control group design/ (329)

292 (pre- adj3 post-).tw,kw. (148690)

293 (pretest adj3 posttest).tw,kw. (7872)

294 controlled study/ (5179828)

295 (control* adj2 stud$3).tw,kw. (417335)

296 Control Groups/ (258423)

297 control group/ (258423)

298 trial.ti. (356638)

299 or/283-298 (6664778)

300 264 and 299 [CCTS/NON-RCTS] (9289)

301 cohort analysis/ (499956)

302 cohort?.tw,kw. (996615)

303 retrospective study/ (1102394)

304 longitudinal study/ (206733)

305 prospective study/ (808493)

306 (longitudinal or prospective or retrospective).tw,kw. (2225517)

307 follow up/ (1170232)

308 ((followup or follow-up) adj (study or studies)).tw,kw. (91372)

309 observational study/ (147400)

310 (observation$2 adj (study or studies)).tw,kw. (169328)

311 population research/ (87628)

312 ((population or population-based) adj (study or studies or analys#s)).tw,kw. (38443)

313 ((multidimensional or multi-dimensional) adj (study or studies)).tw,kw. (185)

314 exp comparative study/ (2690197)

315 ((comparative or comparison) adj (study or studies)).tw,kw. (179471)

316 exp case control study/ (955328)

317 ((case-control* or case-based or case-comparison) adj (study or studies)).tw,kw. (186400)

318 cross sectional study/ (428966)

319 ((crosssection* or cross-section*) adj (study or studies or survey?)).tw,kw. (288696)

320 or/301-319 (7532633)

321 264 and 320 [OBSERVATIONAL STUDIES] (10720)

322 275 or 282 or 300 or 321 [ALL STUDY DESIGNS] (18628)

323 322 use emed (15245) [EMBASE RECORDS]

324 158 or 323 (19552) [BOTH DATABASES]

325 112 use ppez (449)

326 275 use emed (793)

327 325 or 326 (1242)

328 remove duplicates from 327 (986) [UNIQUE REVIEWS]

329 120 use ppez (1979)

330 282 use emed (5921)

331 329 or 330 (7900)

332 331 not 327 (7233)

333 limit 332 to yr="2010-CURRENT" (5618)

334 remove duplicates from 333 (4580)

335 332 not 333 (1615)

336 remove duplicates from 335 (1203)

337 334 or 336 [UNIQUE RCTS] (5783)

338 139 use ppez (871)

339 300 use emed (8229)

340 338 or 339 (9100)

341 340 not (331 or 327) (4857)

342 remove duplicates from 341 [UNIQUE NON-RCTS/CCTS] (4554)

343 156 use ppez (2751)

344 321 use emed (8356)

345 343 or 344 (11107)

346 345 not (327 or 331 or 340) (6220)

347 limit 346 to yr="2010-current" (4884)

348 remove duplicates from 347 (4260)

349 346 not 347 (1336)

350 remove duplicates from 349 (1146)

351 348 or 350 [UNIQUE OBSERVATIONAL STUDIES] (5406)

352 328 or 337 or 342 or 351 (16729)

353 328 use ppez (427) [UNIQUE MEDLINE REVIEWS]

354 328 use emed (559) [UNIQUE EMBASE REVIEWS]

355 337 use ppez (1673) [UNIQUE MEDLINE RCTS]

356 337 use emed (4110) [UNIQUE EMBASE RCTS]

357 342 use ppez (175) [UNIQUE MEDLINE NON-RCTS]

358 342 use emed (4379) [UNIQUE EMBASE NON-RCTS]

359 351 use ppez (1813) [UNIQUE MEDLINE OBSERVATIONAL STUDIES]

360 351 use emed (3593) [UNIQUE EMBASE OBSERVATIONAL STUDIES]

361 352 use ppez (4088) [UNIQUE MEDLINE RECORDS – ALL STUDY DESIGNS]

362 352 use emed (12641) [UNIQUE EMBASE RECORDS – ALL STUDY DESIGNS]

***************************

Cochrane Library

Search Name: HER2 Breast Cancer - Herceptin - Final

Date Run: 19/10/16 23:49:07.686

Description: Cornerstone Research - 2016 Sep 9

ID Search Hits

#1 [mh "Breast Neoplasms"] 9857

#2 ((breast* or mamma or mammary) near/3 (adenocarcinoma* or cancer* or carcinoma* or neoplasm* or tumour* or tumor*)):ti,ab,kw 22260

#3 #1 or #2 22260

#4 [mh "Receptor, ErbB-2"] 596

#5 (ErbB2 or "ErbB 2" or HER2* or (HER next 2*) or "c-ErbB2" or "C-ErbB 2"):ti,ab,kw 4206

#6 ((oncoprotein* or onco-protein* or protein* or receptor*) near/1 (neu or neuregulin)):ti,ab,kw 23

#7 (CD340 near/1 antigen*):ti,ab,kw 0

#8 ("p185(c-neu)" or p185erbB):ti,ab,kw 1

#9 ("neu proto-oncogene" next protein*):ti,ab,kw 0

#10 ("metastatic lymph node gene 19" next protein*):ti,ab,kw 0

#11 "luminal b":ti,ab,kw 69

#12 (luminal next subtype*):ti,ab,kw 17

#13 (human near/1 "epidermal growth factor receptor 2"):ti,ab,kw 298

#14 [mh "Receptor, Epidermal Growth Factor"] Publication Year from 2006 to 2007 42

#15 {or #4-#14} 4289

#16 #3 and #15 2064

#17 [mh ^"Combined Modality Therapy"] Publication Year from 1984 to 1991 1959

#18 [mh "Chemotherapy, Adjuvant"] 3724

#19 ((chemotherap* or chemo-therap*) near/5 (adjuvant* or adjuvent* or neoadjuvant* or neo-adjuvant* or neoadjuvent* or neo-adjuvent* or adjunct* or neoadjunct* or neo-adjunct*)):ti,ab,kw 8121

#20 (((drug next therap*) or pharmacothera* or pharmaco-therap*) near/5 (adjuvant* or adjuvent* or neoadjuvant* or neo-adjuvant* or neoadjuvent* or neo-adjuvent* or adjunct* or neoadjunct* or neo-adjunct*)):ti,ab,kw 667

#21 [mh "Breast Neoplasms"/DT] 4385

#22 [mh "Antineoplastic Agents"] 11658

#23 ("anti-HER2" or "anti-HER 2"):ti,ab,kw 111

#24 ((HER2 or "HER 2") near/1 block*):ti,ab,kw 20

#25 ((HER2 or "HER 2") near/1 antagoni*):ti,ab,kw 0

#26 [mh "Receptor, ErbB-2"/AI] 56

#27 [mh "Antineoplastic Combined Chemotherapy Protocols"] 12366

#28 [mh Trastuzumab] 212

#29 (herceptin* or trastuzumab* or TZM):ti,ab,kw 1092

#30 ((ado next trastuzumab*) or kadcyla or "pro 132365" or pro132365 or "t dm 1" or "t dm1" or "tmab mcc dm1"):ti,ab,kw 35

#31 [mh Cyclophosphamide] 4096

#32 (alkyroxan or "b 518" or b518 or carloxan or ciclofosfamida or ciclolen or cicloxal or clafen or "cyclo-cell" or cycloblastin or cycloblastine or cyclofosamide or cyclofosfamid or cyclofosfamide or cyclophar or cyclophosphamide or cyclophosphamides or cyclophosphan or cyclophosphane or cyclostin or cycloxan or cyphos or cytophosphan or cytophosphane or cytoxan or endocyclophosphate or endoxan or endoxana or enduxan or genoxal or ledoxan or ledoxina or mitoxan or neosan or neosar or noristan or "nsc 26271" or nsc26271 or "nsc 2671" or nsc2671 or procytox or procytoxide or semdoxan or sendoxan or syklofosfamid):ti,ab,kw 8235

#33 [mh Doxorubicin] 3585

#34 (adriablastin* or adriacin or adriamicin* or adriamycin* or adriblastin* or adrim or adrimedac or adrubicin or amminac or caelix or caelyx or carcinocin or "dox sl" or doxil or "DOXO-cell" or doxolem or "doxor lyo" or doxorubicin* or doxotec or evacet or farmiblastina or "fi 106" or fi106 or ifadox or lipodox or "mcc 465" or mcc465 or myocet* or "nsc 123127" or nsc123127 or onkodox or rastocin* or resmycin or ribodoxo or rubex or rubidox or sarcodoxome or "tlc d 99"):ti,ab,kw 6112

#35 [mh Epirubicin] 838

#36 ("4'-Epi-Adriamycin" or "4'-Epi-Doxorubicin" or "4'-Epi-DXR" or "4'-Epiadriamycin" or "4'-Epidoxorubicin" or binarin or ellence or "EPI-cell" or epiadriamycin or epidoxo or epidoxorubicin or epidx or epifil or epilem or epirubicin or farmorrubicina or farmorubicin* or "IMI-28" or "NSC-256942" or NSC256942 or pharmorubicin* or pidorubicin):ti,ab,kw 2231

#37 [mh Fluorouracil] 4282

#38 ("5-FU" or 5FU or "5-fluorouracil" or 5fluorouracil or "5-fluoruracil" or 5fluoruracil or accusite or actino-hermal or adrucil or carac or effluderm or efudex or efudix or efurix or f6627 or fivoflu or "fluoro-uracile" or fluoroblastin or fluoroplex or fluorouracil or fluoruracil or fluouracil or fluracedyl or flurodex or fluracil or fluracilium or fluril or fluroblastin or flurouracil or fluoxan or "haemato-fu" or ifacil or neofluor or "nsc 18913" or nsc18913 or "nsc 19893" or nsc19893 or oncofu or onkofluor or ribofluor or uflahex or utoral or verrumal):ti,ab,kw 8513

#39 [mh Capecitabine] 379

#40 (apecitab or ecansya or capecitabine or xeloda):ti,ab,kw 1525

#41 [mh Methotrexate] 3093

#42 (amethopterine or abitrexate or amethopterin or amethopterine or ametopterine or antifolan or biotrexate or canceren or "cl 14377" or cl4377 or emtexate or emthexat or emthexate or emtrexate or enthexate or farmitrexat or farmitrexate or farmotrex or folex or ifamet or imeth or "intradose MTX" or lantarel or ledertrexate or maxtrex or metex or methoblastin or methohexate or methotrate or methotrexat or methotrexate or methotrexato or methoxtrexate or methrotrexate or methylaminopterin or methylaminopterine or meticil or metoject or metothrexate or metotrexat or metotrexate or metotrexin or metrex or mexate or "mpi 5004" or mpi5004 or neotrexate or novatrex or "nsc 740" or nsc740 or otrexup or rasuvo or reumatrex or rheumatrex or texate or texate-t or texorate or trexall or xaken or zexate):ti,ab,kw 6870

#43 gemcitabine:ti,ab,kw 2399

#44 (gemcite or gemzar or "ly 188011" or ly188011):ti,ab,kw 29

#45 [mh Carboplatin] 1134

#46 (blastocarb or boplatex or carboplat or carboplatin or carboplatino or carbosin or carbotec or carplan or CBDCA or cycloplatin or erbakar or ercar or ifacap or kemocarb or "nsc 241240" or nsc241240 or oncocarbin or paraplatin or paraplatine):ti,ab,kw 3262

#47 [mh Cisplatin] 3586

#48 (abiplatin or biocisplatinum or biocysplatinum or blastolem or briplatin or "cis ddp" or cis diamine dichloroplatinum or cis diaminechloroplatinum or cis diaminedichloroplatinum or cis diammine dichloroplatinum or cis diamminedichloroplatinum or cis dichloridiammineplatinum or cis dichloroadiamine platinum or cis dichlorodiamine platinum or cis dichlorodiamineplatinum or cis dichlorodiammine platinum or cis dichlorodiammineplatinum or cis platinous diamino dichloride or cis platinum or cisplatin or cisplatine or cisplatino or cisplatinum or cisplatyl or citoplatino or cytoplatin or cytosplat):ti,ab,kw 8741

#49 (diamine dichloroplatinum or diaminodichloroplatinum or diamminedichloroplatinum or dichlorodiamine platinum or dichlorodiammineplatinum or docistin or elvecis or kemoplat or lederplatin or lipoplatin or "mpi 5010" or mpi5010 or neoplatin or niyaplat or "nk 801" or nk801 or noveldexis or "nsc 119875" or nsc119875 or platamine or platiblastin or platidiam or platimine or platinex or platinil or platinol or platinoxan or platiran or platistil or platistin or platosin or randa or romcis or sicatem or "spi 077" or tecnoplatin):ti,ab,kw 104

#50 (platinum* near/1 (diaminodichloride or "diamino dichloride" or "diamine dichloride" or diaminedichloride or diaminodichloride or diamminedichloride)):ti,ab,kw 2

#51 docetaxel:ti,ab,kw 3142

#52 (daxotel or dexotel or docefrez or "lit 976" or lit976 or "nsc 628503" or nsc628503 or oncodocel or taxoter or taxotere or texot):ti,ab,kw 189

#53 [mh Paclitaxel] 1747

#54 ("abi 007" or abi007 or abraxane or anzatax or asotax or biotax or "bms 181339" or bms181339 or bristaxol or britaxol or coroxane or formoxol or genexol or hunxol or ifaxol or infinnium or intaxel or "mbt 0206" or mbt0206 or medixel or mitotax or "nsc 125973" or nsc125973 or oncogel or onxol or pacitaxel or pacxel or padexol or parexel or paxceed or paxene or paxus or praxel or taxocris or taxol or taxus or taycovit or yewtaxan):ti,ab,kw 638

#55 vinorelbine:ti,ab,kw 811

#56 ("anx 530" or anx530 or eunades or exelbine or "kw 2307" or kw2307 or navelbin or navelbine or navirel or vinbine or vinelbine):ti,ab,kw 478

#57 [mh Bevacizumab] 624

#58 (altuzan or avastin or bevacizumab or "nsc 704865" or "nsc704865"):ti,ab,kw 2173

#59 lapatinib:ti,ab,kw 368

#60 ("gw 2016" or gw2016 or "gw 572016" or gw572016 or "gw 572016f" or gw572016f or tykerb or tyver):ti,ab,kw 25

#61 pertuzumab:ti,ab,kw 114

#62 ("monoclonal antibody 2C4" or omnitarg or perjeta or "rhumab 2C4"):ti,ab,kw 6

#63 neratinib:ti,ab,kw 19

#64 ("HKI 272" or HKI272 or "way 177820" or way177820):ti,ab,kw 3

#65 anastrozole:ti,ab,kw 687

#66 (arimidex or "ici d1033" or icid1033 or trozolet or "ZD-1033" or ZD1033):ti,ab,kw 171

#67 exemestane:ti,ab,kw 477

#68 (aromasil or aromasin or aromasine or "FCE 24304" or nikidess or "pnu 155971" or pnu155971):ti,ab,kw 22

#69 fulvestrant:ti,ab,kw 190

#70 (faslodex or "ICI 182,780" or "ICI 182780" or "zd 182780" or zd182780 or "zd 9238" or zd9238 or "zm 182780" or zm182780):ti,ab,kw 63

#71 letrozole:ti,ab,kw 867

#72 ("CGS 20267" or CGS20267 or femar or femara):ti,ab,kw 35

#73 [mh Tamoxifen] 1995

#74 ("ICI-46,474" or "ICI-46474" or "ICI-47699" or kessar or nolvadex or novaldex or "nsc 180973" or nsc180973 or soltamox or tamoplac or tamoxasta or tamoxifene or tomaxithen or zitazonium):ti,ab,kw 101

#75 {or #17-#74} 51148

#76 #16 and #75 Publication Year from 1990 to 2016 1709

DSR - 7

DARE - 40

CENTRAL – 1584

Methods – 5 [*did not download*]

HTA - 41

NHS EED – 32 [*did not download*]
